# Notes about the uses of plants by one of the last healers in the Basilicata Region (South Italy)

**DOI:** 10.1186/1746-4269-8-15

**Published:** 2012-04-30

**Authors:** Vincenzo Montesano, Donatella Negro, Giulio Sarli, Antonino De Lisi, Gaetano Laghetti, Karl Hammer

**Affiliations:** 1 National Research Council, Institute of Plant Genetics, Via Amendola, 165/A, Bari, 70125, Italy; 2 Institute of Crop Science, former Professor of Agrobiodiversity c/o University of Kassel, Witzenhausen, Germany

**Keywords:** Traditional healer, *Tamarix gallica*, Basilicata region (Italy)

## Abstract

**Background:**

The paper refers to the knowledge and uses of plants and to the linked ritual practices as referred by Matteo (It.*‘Zì Matteo’,* En. *‘Uncle Matthew’*), one of the last elder healers in the Basilicata Region (South Italy). Particular attention is also paid to the uses of *‘Vruca’* (*Tamarix gallica* L.) as a medicinal and magical plant used to heal common warts on various parts of the body.

**Methods:**

After obtaining prior informed consent, we collected data through an open interview about the uses of the plants and on the associated ritual practices. For each species, data were collected that included the vernacular names, preparation, plant parts utilized and their method of use.

**Results:**

The uses of 52 taxa are described. Among these, 43 are or were employed medicinally, eight as culinary foodstuffs, and 4 for domestic, handicraft or ethnoveterinary uses.

Among the major findings: the ritual and magical use of *Tamarix gallica* L. to heal warts is described in detail; so far, no records of similar use were found in any Italian ethnobotanical studies conducted in southern Italy.

**Conclusion:**

Phytotherapy in the Basilicata region is practiced by elderly people who resort to medicinal plants for mild illnesses; we interviewed one of those traditional healers who is very experienced in the field, and possesses rich ethno-pharmacological knowledge.

## Background

Traditional Healing (TH) is the oldest form of structured medicine, and was originally an integral part of semi-nomadic and agricultural tribal societies; although archaeological evidence for its existence dates back to only around 6000 B.P., its origins probably date back to well before the end of the last Ice Age [1]. There were and still are differences between the principles and philosophy of TH, although there are also many fundamental similarities that arise from the profound knowledge of natural laws, and the understanding of how these influence living things, that are shared by all Traditional Healers (THs). THs are found in most societies and are often part of a local community, culture and tradition, and they continue to have high social standing in many places, exerting influence on local health practices.

**Figure 1:**
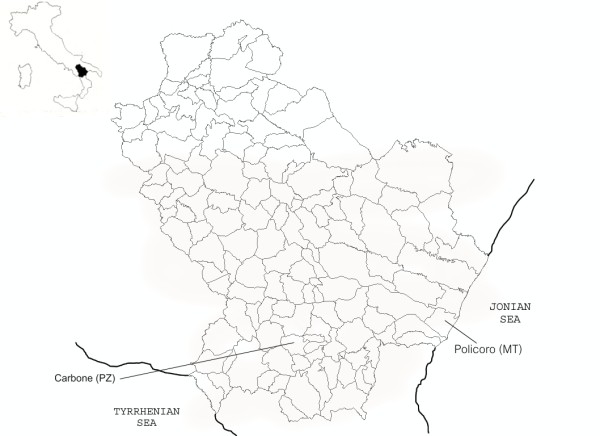
geographical position of the Basilicata (Lucania) region with indicate the towns of Carbone (in province of Potenza) and Policoro (in province of Matera).

In the Basilicata region (Figure 1) (once known as Lucania), as is generally true throughout southern Italy, the traditional (magic) healing is a belief that fascinates and intrigues, and has always held a position of considerable importance in the daily events of life. Common people have a sense of respect and awe for the mysterious powers the THs possess, since they are the only and absolute keepers of “mysterious forces” that can be used *ad libitum*. The respect and awe they inspire find their external manifestation in the titles of deference, friendship, and sometimes empathy given them (e.g.*“Zi,”* meaning *“Uncle”* in English, or *“Compare,”* meaning *“good friend”* in English), these special relationships being due entirely to their special abilities.

Nowadays these THs are disappearing, especially in industrialized countries, because their knowledge ends with their death. Typically they are a very intelligent men or women who own the “powers” and that, targeting to a person’s psyche, can very often subjugate their own will. Because of the trend of migration out of the countryside and away from agricultural activities, today only vestiges of this knowledge survive, and that only amongst farmers, shepherds and elderly people.

The general isolation of the Basilicata region, and of the mountainous areas in particular -still partially based on small-scale agricultural and pastoral activities (raising sheep and to decreasing extent the “Podolica” cattle)- make it very conducive to studies of local traditional pharmaceutical knowledge (TPhK) [2] and (ethno)botany [3,4].

In this region some ethnobotanical studies have been conducted over the last 30 years [2-21], almost all of them based mainly on interviews with farmers, shepherds and housewives who are either native to the area or have been living there since childhood, most of them over 60 years old; but few have been carried out with healers *sensu strictu*. It is apparent that knowledge of traditional natural remedies for healing human diseases is disappearing in Lucania: modern pharmaceuticals have replaced many natural remedies, and no real healers remain in most areas of the region.

In this paper we study the ethnopharmacy and TPhK of ‘*Zì Matteo’* (*‘Uncle Matthew’*), one of the last elder healers still living and recognised by the local population.

He is a native of Carbone, a small town in the inland areas of Basilicata, and after the land reform of the 60s -which led to the reclamation of coastal wetlands, allowing the development of intensive agriculture on this land- he moved with his family to Policoro (MT) (Figure 1), where he worked as a farmer and where he still lives.

At this time he is considered to be one of the most respectable and popular healer in the area. He inherited almost all of his knowledge when he was a boy, from a healer who was old and dying, and continued the pursuit with the suggestions of *‘Zi Giuseppe’* (‘*Uncle Joseph’*), perhaps the most popular healer in the province of Matera, who lived in Valsinni (a village a few kilometers away).

## Methods

The fieldwork was conducted over a period of five months, from November 2010 to March 2011, and took place in Policoro (MT), where the widower Matteo, 87 years old, lives *“the last years of his life”*, as he many times repeated.

On an anthropological level, the Basilicata area is known for having been described in the late 1950s by the Italian anthropologist Ernesto de Martino, who lived in the region during the spring of 1957 [22,23].

In our study, after obtaining prior informed consent, and using standard methods [24,25], we collected data about the uses of the plants and the linked ritual practices. Ethnobotanical information was collected using open and semi-structured interviews to *‘Zi Matteo’* and also to another 10 persons chosen at random among the elderly population of the area (Policoro and near towns), who still retain traditional knowledge about medicinal plants. For each species, data were collected including plant parts utilized, their preparation, and their method of use.

Women have generally retained the most information concerning traditional domestic remedies, and therefore the interviewees were more heavily female: seven women and three men, with average age of 69.

Information was gathered through observation either of the present use of traditional plant pharmaceuticals, or of uses that are at least still alive in the collective memory of the oldest segment of the population.

During the interviews, several fresh plant specimens or dried samples, stocked in a small transportable field herbarium, were shown to the interviewees. Each non-cultivated botanical species recognized by the study participants as having been used for medicinal purposes was collected and identified by the authors; the voucher material will be sent to the *Herbarium Lucanum* (HLUC) in Potenza, Italy.

The transcription of vernacular names of the recorded traditional remedies of the local folk pharmacopoeia follow the rules of the Italian language, and non-domesticated medicinal plants were identified following the Italian botanical standard treatise [26], whereas cultivated plants were identified using Hammer et al [27]. All the scientific names of the plants were confirmed through Tropicos database [28].

## Results and discussion

Table 1 reports the ‘traditional’ plant remedies used until recently by *‘Zi Matteo’* and confirmed by other interviewees: reported in this case were only the plants whose uses were quoted by at least 70% of informants (calculated as percentage of all interviewees spontaneously quoting a given remedy). They represent the traditional heritage of the ethnopharmacopoeia of this part of the region.

**Table 1:** **Uses of traditional plant phytotherapeuticals recorded during the interview to*‘Zi Matteo’*and confirmed by other participants (Legend: Status: C: cultivated, SC: semi-cultivated, W: wild; Use(s): M: medicinal uses, E: ethnoveterinary uses, F: food uses, D: domestic and handicraft uses)**

**Botanical taxon**	**Botanical family**	**Vernacular name recorded**	**Status**	**Part(s) utilized**	**Preparation**	**Use(s)**	**Claimed use(s)**
*Agave americana* L.	Asparagaceae	*sciabblon*	W	Leaves	Decoction is left overnight and then applied topically.	M	Treatment of muscle and rheumatic pain
*Allium cepa* L.	Amaryllidaceae	*cipudda*	C	Bulbs	Oleolite (frying)	M	Healing of purulent skin abscesses (caused by thorns)
					Soup	M	Galactagogue
					Crushed and then applied topically	M	Treatment of bruises
					Used as an aromatic in the kitchen	F	Spice
*Allium sativum* L.	Amaryllidaceae	*dd’agghie*	C	Bulbs	Eaten raw	M	Anti-hypertensive
					Ground, mixed with parsley, and applied topically	M	Healing of insect bites
					Necklace, to be worn by babies or children	M	Vermifuge
					Heated on hot charcoal, then applied topically	M	Healing of warts
					Used as an aromatic in the kitchen	F	Spice, aromatizing sausages
*Aloysia citrodora* Palau	Verbenaceae	*citronella*	C, SC	Leaves	Streaked on the skin	M	Mosquito repellant
*Amaranthus retroflexus* L.	Amaranthaceae	*p’pon salvagge*	W	Young buds	Picked before flowering	F	Food
*Anethum graveolens* L.	Apiaceae	*f’nucchiastr*	C	Leaves	Picked before flowering	F	Flavoring for canned olives
				Seeds	Used as an aromatic in the kitchen	F	Spice for sausages and for Easter cakes
*Avena sativa* L.	Poaceae	*biama/avena*	C	Seeds	Decoction	M	Reconstituent for small children
*Arundo donax* L.	Poaceae	*canna*	W	Membrane	Topical application	M	Haemostatic
*Arum italicum* Mill.	Araceae	*‘nzale*	W	Sap	Topical application	M	Healing of warts
*Asparagus acutifolius* L.	Asparagaceae	*sparije*	W	Shoots	Boiled and consumed alone or with scrambled eggs and fresh cheese	F	Diuretic
*Borago officinalis* L.	Boraginaceae	*vurrajiene*	W	Aerial parts (at flowering)	Soup prepared with *Allium cepa*	F	Galactagogue
					Decoction	M	Sore throat remedy
					In salad (finely minced leaves)	F	Food
*Brassica oleracea* L.	Brassicaceae	*cav’l*	C	Leaves	Roasted and applied topically	E	Treatment of mastitis or shoulder pain
*Calamintha nepeta* L.	Lamiaceae	*nepita*	W	Leaves	Decoction for local washing	E	Treatment of rhagades and mastitis of cattle
*Capsicum annuum* L.	Solanaceae	*cancaricchi’e*	C	Pods	Used as an aromatic in the kitchen	F	Anti-hypertensive
				Seeds	Fitted in a little bag and attached to clothing as an amulet (ritual-medical use)	M	Treatment of evil-eye
*Ceratonia siliqua* L.	Leguminosae	*carrubba*	C	Fruits	Decoction with *Ficus carica* L and *Malva sylvestris* L	M	Emollient
*Cichorium intybus* L.	Asteraceae	*cicoria catalogna*	C	Aerial parts	Soup	M	Laxative
*Citrus limonum* Risso	Rutaceae	*l’mon’e*	C	Fruits	Decoction	M	Treatment of diarrhea
*Citrus sinensis* (L.) Osb.	Rutaceae	*purt’galle*	C	Fruits	Decoction in mixtures with other species	M	Treatment of sore throat and cough
*Clematis vitalba* L.	Ranunculaceae	*vitacchia*	W	Fruits	Decoction used as a gargle	M	Healing of mouth inflammations
*Cynara scolymus* L.	Asteraceae	*scaler’*	W	Flower heads	Decoction	M	Liver depurative
*Cynodon dactylon* (L.) Pers.	Poaceae	*gramigna*	W	Rhizome	Decoction	M	Diuretic
*Diplotaxis tenuifolia* (L) DC.	Brassicaceae	*ruca salvagg’e*	W	Leaves	Oleolite (fried leaves) applied topically	M	Treatment of muscle pain
*Ecballium elaterium* (L.) A. Rich.	Cucurbitaceae	*cucc’ marrong’e*	W	Fruits	Decoction used as a gargle	M	Toothache remedy
*Eucalyptus globulus* Labill.	Myrtaceae	*calipso*	C, SC	Leaves	Decoction with *Malva sylvestris*	M	Respiratory tract antiseptic
*Ficus carica* L.	Moraceae	*fica*	C, SC	Fruits	Dried, then decoction with other herbs	M	Treatment of sore throats and bronchitis, intestinal depurative
				Sap	Topical application	M	Healing of insect bites and warts
*Glycyrrhiza glabra* L.	Leguminosae	*pruvlizije*	SC	Roots	Decoction	M	Anti-tussive agent
*Hordeum vulgare* L.	Poaceae	*dd’orije*	C	Seeds	Decoction, in mixtures with other vegetables	M	Treatment of sore throats and bronchitis
					Bread embedded with a decoction of the seeds	M	Reconstituent for ill children and elderly
*Juglans regia* L.	Juglandaceae	*nucia*	C, SC	Leaves	Leaves put into the shoes	M	Treatment of excessive foot perspiration
*Lactuca sativa* L.	Asteraceae	*lattuca*	C	Leaves	Decoction used as a gargle and a topical application	M	Treatment of gingival abscess
					Boiled, applied topically	M	Toothache remedy
*Laurus nobilis* L.	Lauraceae	*laur’*	SC	Leaves	Decoction	M	Treatment of sore throat and stress
*Malva sylvestris* L.	Malvaceae	*malva*	W	Aerial parts (at flowering)	Decoction	M	Mild laxative
					Decoction	M	Treatment of furuncles, abscesses and dermatitis (in babies)
					Fresh leaves are boiled and placed into cloth sack to use as a warm compress	E	Treatment of mastitis
*Marrubium vulgare* L.	Lamiaceae	*marrug’*	W	Aerial parts (at flowering)	Decoction used as a wash	M	Healing of cysts, panacea
*Myrtus communis* L.	Myrtaceae	*murtedda*	W	Fruits	Infusion	M	Treatment of colds
					Ash sprinkled into shoes	M	Treatment of excessive foot perspiration
*Papaver somniferum* L.	Papaveraceae	*papagna*	SC	Fruits	Decoction	M	Tranquillizer
*Petroselinum crispum* (Mill.) A.W. Hill	Apiaceae	*putrsin’*	C	Aerial parts	Decoction	M	Abortion inducement
					Ground with garlic (*Allium sativum*) bulbs and topically applied	M	Healing of insect bites
					Decoction with fern roots	M	Abortion inducement
*Prunus domestica* L.	Rosaceae	*prugn*	C	Fruits	Dried and eaten	M	Laxative
*Pyrus communis* L.	Rosaceae	*pir’*	C	Fruits	Boiled	M	Mild laxative
*Ocimum basilicum* L.	Lamiaceae	*basilic*	C	Leaves	Smell inhaled	M	Headache remedy
*Olea europaea* L.	Oleaceae	*guliv’*	C	Leaves	Leaves chewed	M	Stomachache remedy
*Origanum vulgare* L. ssp. *viride* (Boiss.) Hayek	Lamiaceae	*rigan’e*	W	Aerial parts (at flowering)	Smoked, fumes inhaled	M	Toothache remedy
*Populus alba* L.	Salicaceae	*chiupp*	C, SC	Young stems	Handcrafted	D	Collars for cows
*Portulaca oleracea* L.	Portulacaceae	*purchiazza*	SC	Aerial parts	In salad or boiled	F	Food
*Rosmarinus officinalis* L.	Lamiaceae	*ros’marin*	C	Aerial parts (at flowering)	Decoction	M	Sore throat or stomachache remedy
*Ruta graveolens* L.	Rutaceae	*ruta*	C	Aerial parts (at flowering)	Oleolite(fried) applied topically	M	Treatment of muscle pain
*Salvia officinalis* L.	Lamiaceae	*salvia*	C	Aerial parts (at flowering)	Decoction	M	Sore throat remedy
				Leaves	Rubbed on the teeth	M	Teeth-whitener
*Salix alba* L.	Salicaceae	*salic*	SC, W	Young stems	Handcrafted	D	Tying vines
*Sambucus nigra* L.	Adoxaceae	*sambuc*	W	Fruits	Decoction	M	Diaphoretic
					Decoction with other species	M	Sore throat remedy
					Squashed on the skin	M	Healing of insect bites
*Silybum marianum* (L.) Gaert.	Asteraceae	*cardon’*	W	Aerial parts	In salad or cooked	F	Food
					Boiled with other vegetables	F	Food
*Spartium junceum* L.	Fabaceae	*g’nestra*	W	Stems	Topical application	M	Healing of warts
*Tamarix gallica* L.	Tamaricaceae	*vruc/tamarice*	W	Young stems	Ritual-magical use	M	Healing of warts
*Taraxacum officinale* Weber	Asteraceae	*cicorion’*	W	Aerial parts	In mixed salad or as boiled vegetable	F	Food
*Urtica dioica* L.	Urticaceae	*r’ddicula*	W	Aerial parts	Decoction	M	Digestive agent
					Decoction	M	Liver protection
					Local washing	M	Treatment of hemorrhoids
					Boiled leaves mixed with tomatoes and eaten	M	Treatment of renal problems
					Cooked (e.g. in omelettes, croquettes, and soups)	F	Mineralizing

In this table, we reported for each botanical taxon (or remedy) its botanical family, folk (vernacular) name(s), its conservation status (C: cultivated; SC: semi-cultivated; W: wild ), the utilized parts, the means of administration/preparation, the claimed uses (M: medicinal; E: ethnoveterinary; F: food; D: domestic and handicraft), and the observation of a claimed use recorded during the field study.

The uses of 52 plants belonging to 30 families are reported: two taxa are employed in domestic and handicraft uses, two for ethnoveterinary, eight for food and 40 for medicinal uses (Table 1). The most highly represented families are Lamiaceae (6 species), Asteraceae (5 species), Poaceae (4 species) and Rutaceae (3 species). Plants are listed according to alphabetical order of their botanical names. Seventy-nine remedies of plant origin (belonging to 52 botanical taxa) were recorded.

The parts of the plants most used for medicinal purposes in decreasing order are leaves and aerial parts, fruits, seeds, young stems, bulbs and sap, flower heads, membrane, pods, rhizome and roots, shoots and stems, and young buds. External uses predominate over internal ones by about 54 to 46%. Decoction, almost always in water, is the main method of preparation for oral administration, while direct application of the plants is the most important method for topical use.

Most pathologies treated with plants are dermatological (47%), respiratory (19.3%), headache, toothache, or muscular pain (17.7%), digestive (15%) and renal (1%).

As for the food uses, the collection of wild edible plants took place during work in the fields. Occasionally the plants were eaten raw as a snack by the workers, or were brought home and cooked in a traditional terracotta pot. In the poorest years of his life (when he was young), *‘Zi Matteo’* reports that these wild greens were eaten raw with bread, without olive oil or salt. Today only a few wild edibles are consumed in their raw form; instead, they are commonly prepared by light boiling, or frying in olive oil, with garlic, salt, and sometimes a few hot chili peppers, and usually eaten with bread.

The plants reported grow mainly in meadows, ruderal areas, edges of roads, woods, open environments and in the Mediterranean maquis; some species are gathered near rivers or canals, while others are cultivated. As in other parts of the Basilicata region and elsewhere in Italy, several species are eaten (*Allium cepa* L., *Amaranthus retroflexus* L., *Anethum graveolens* L*., Asparagus acutifolius* L*., Borago officinalis* L*., Capsicum annuum* L., *Portulaca oleracea* L*., Silybum marianum* (L.) Gaertn*., Taraxacum officinale* Weber ex Wiggers*)*. Among the less common food uses we cite is that of the young buds of *Amaranthus retroflexus*, a weedy or ruderal species. Some thorny plants (*Silybum marianum*) are also eaten in other parts of the region. Among the seasoning herbs, we cite *Myrtus communis* L*., Laurus nobilis* L.*, Origanum vulgare* L. ssp. *viride* (Boiss.) Hayek and *Salvia officinalis* L*.*

Concerning the transmission of cultural or traditional knowledge about cultivated or wild plants, in the opinion of *‘Zi Matteo’* this mechanism has broken down: young people no longer go into the fields, but instead work at home or in factories in the surrounding areas, and do not gather wild edibles, but cure their health problems instead with commercial drugs.

### The story of *‘Zi Matteo’* and the use of *Tamarix gallica* L

Usually skills and traditional knowledge are transferred from grandparents to their grandchildren; it is common practice for grandchildren to accompany their grandparents during the collection of medicinal plants and during the treatment of patients. Through these interactions, children become interested in this profession, and may be motivated to practice it.

Nevertheless, in this study *‘Zi Matteo’* indicates that he became a healer through another healer (‘*Zi Giuseppe’*), indicating that a form of initiation took place. He did not communicate how exactly this interaction was initiated and which form it took; he reported only that he was led to *‘Zi Giuseppe’* by his father’s suffering from pain. Since then, he began to be trained in the uses of the plants and the rituals linked to the treatments. At this point it is important to emphasize that, despite his moving from the inland native area to another place in the region with slightly different environmental conditions, his ethnobotanical knowledge was not significantly changed, by his estimation.

During this time he also learned how to prevent illnesses, especially the evil-eye (called ‘*affascene’*), and he described to us the relevance of the ‘evil-eye’ system in this part of the region (see also Quave and Pieroni [29,30] and Giusti et al [31]). It is also important to note that for *‘Zi Matteo’* it is fundamental not to take money from those who are healed, but only to accept gifts.

Of particular interest, since it has never been quoted or reported before in South Italy, is the magical/ritual use of the young stems of *Tamarix gallica* L. to heal warts on all parts of the body (hands, face, feet, knees, elbows, etc.).

**Figure 2:**
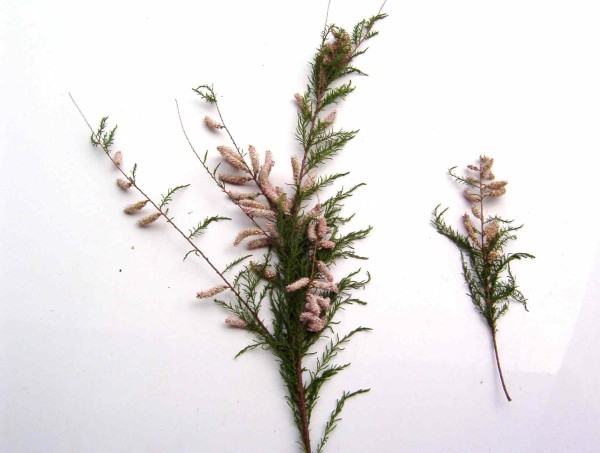
**Young stems of*Tamarix gallica*L. used to heal warts.**

When a person afflicted with warts approaches him, first the healer asks his full name and date of birth, and then sends him home, reassuring him that within a short time the warts will pass. Over the following days, the healer goes along the edges of rivers gathering young stems of the *T. gallica* L. (Figure 2) and brings them home, where he repeats the following sentence for three times:

*“Tamarici, tamarici, tamarici / di NN anni avete / di lunedì siete nato / tamarici AB ha il porro e non me lo dici…”* (In English: “*Tamarisk, tamarisk, tamarisk / NN old you are / you were born on a Monday / tamarisk, AB has the warts and does not tell me..*.”); NN is the age of the person and AB his or her name. While *‘Zi matteo’* casts this “spell”, in his hands he twists the stems of *T. gallica* and then places it on a stone; when this branch has completely dried, the warts disappear.

To our knowledge, amongst the magical and ritual uses of this plant, this practice is recorded here for the first time; however both in Sardinia [32] and Sicily [33] cited in [34] this plant is reported to heal warts or animal infections with other rituals.

## Conclusions

It is apparent that knowledge of traditional natural remedies for healing human diseases is quickly disappearing in this part of the Lucania (Basilicata) Region, in accordance with Pieroni et al [2]. The few people who still retain this knowledge are mostly elderly, and they worry that the chain will soon be broken: modern pharmaceuticals have replaced many natural remedies, and there will someday remain no real healers in the area.

*‘Zi Matteo’* is one of them, and his heritage probably will end up with him, or at best with his heir (his son). Nevertheless, many people still remember the last perceived ‘healer’ of the area, *‘Zi Giuseppe’*, who died many years ago, and is still considered to have been one of the most “powerful” of the Basilicata region; the people interviewed presume that *‘Zi Matteo’* learned most of his knowledge and “powers” from *‘Zi Giuseppe’* alone.

A great heritage in the field of folk ‘domestic medicine’ may still exist, but most of the remedies quoted in this survey have been abandoned, or are rarely in use today. Only a few of them are still used in the primary health care of the family, normally dispensed by the oldest women of the family. Phytotherapy in this small southern Italian region is today practiced by elderly people who resort now only to medicinal plants for mild illnesses; we interviewed one of them and verified the data with others that, although not recognized as “real” healers, are very experienced in the field.

## Competing interests

The authors declare that they have no competing interests.

## Authors’ contributions

VM designed the questionnaire, analyzed and drafted the report on the medicinal plants used by the traditional healer, organized the compiled research data and carried out the analysis of the data, and wrote the article. VM and DN interviewed Matteo and the other elders. GS, ADL, GL and KH participated in writing the paper, and reviewed the data and the whole manuscript. All authors read and approved the final manuscript.
